# Semi-firm lesions on the flanks and extensor surfaces

**DOI:** 10.1016/j.jdcr.2024.01.032

**Published:** 2024-02-16

**Authors:** Crystal T. Chang, Joyce Kim, Heather Carney, Jingyun Gao

**Affiliations:** a Kaiser Permanente Bernard J. Tyson School of Medicine, Pasadena, California; b Department of Dermatology, Kaiser Permanente Los Angeles Medical Center, Los Angeles, California; c Department of Dermatopathology, Mid Atlantic Permanente Medical Group, Rockville, Maryland

**Keywords:** histoid leprosy, keloid, lepromatous, leprosy, mycobacterium leprae, tuberculous leprosy

## History

**Fig 1:**
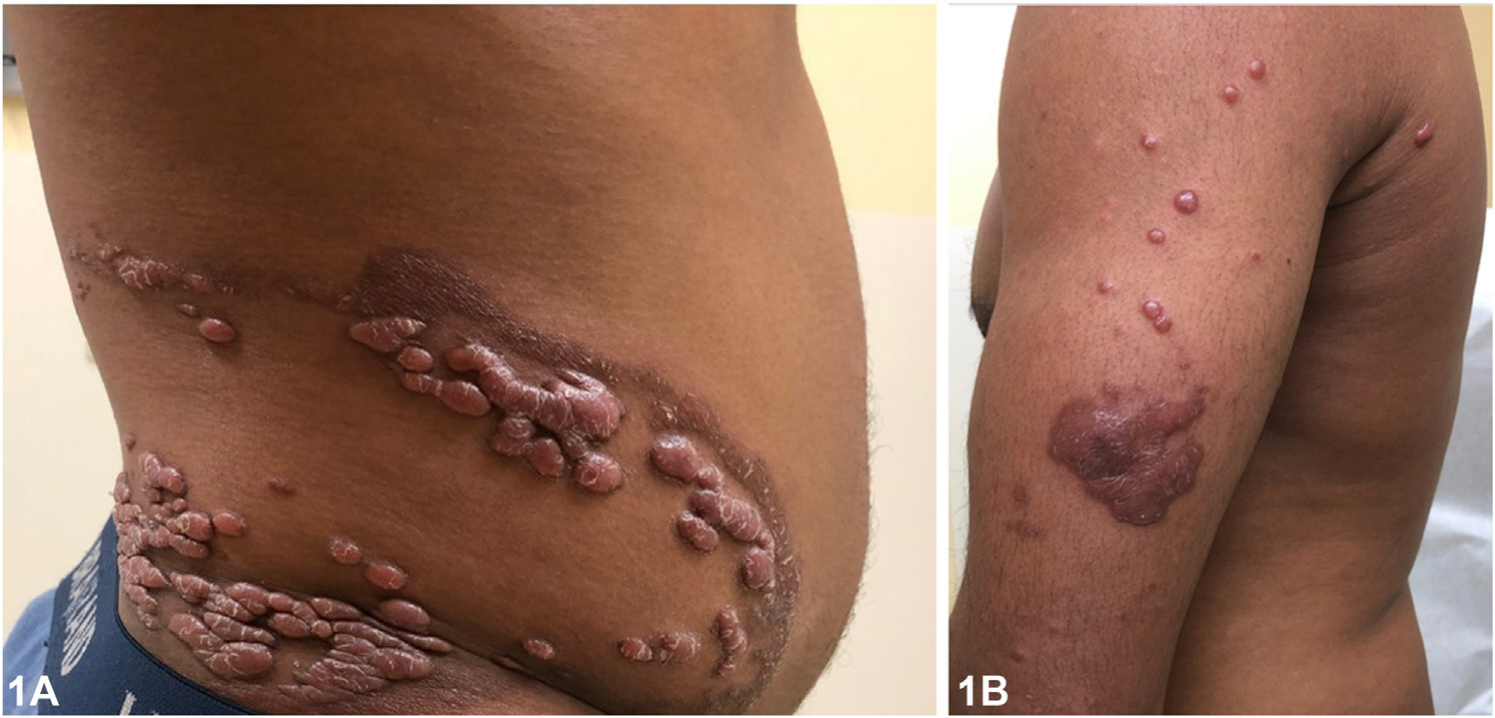

**Fig 2:**
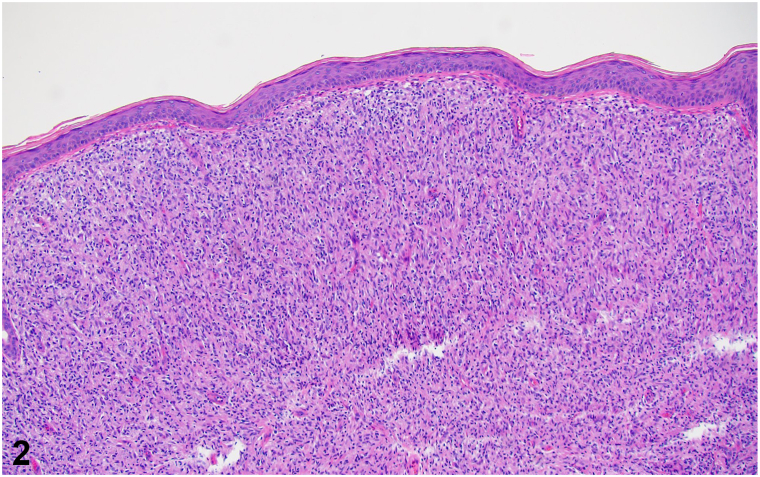

**Fig 3:**
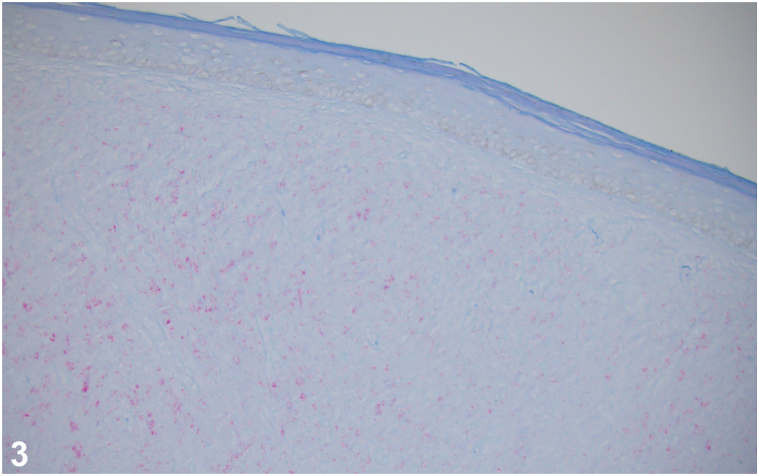

An otherwise healthy Nigerian man in his 20s presents with pruritic lesions that progressed over the past 18 months. He reports the lesions started as a few grouped bumps on the flank that quickly spread over most of the torso. He denies preceding trauma. Due to the COVID-19 pandemic, his initial visit, 6 months prior to his clinic visit, was done through video, when he received fluocinonide ointment for presumed keloids. On exam, pink-brown, semi-firm, dome-shaped papules and nodules were arranged in a curvilinear array along the flank with an advancing scaly border (Fig 1, *A*). Additionally, semi-firm, flat topped plaques with overlying scale were noted on the left dorsal hand, left upper arm (Fig 1, *B*), and ankles. The remaining examination was normal. Shave biopsy of a flank papule was performed (Figs 2 and 3).

**Question 1: Based on this patient’s history and exam findings, what is the most likely diagnosis?**

**A.** Cutaneous lymphoma
**B.** Sarcoidosis
**C.** Keloid scars
**D.** Leprosy
**E.** Leishmaniasis

**Answers:**

**A.** Cutaneous lymphoma – Incorrect. Cutaneous lymphoma can be considered clinically with various clinical presentations, including nodules as seen in this patient. However, the histology of lymphoma would demonstrate large aggregates of atypical lymphocytes rather than granulomatous inflammation with primarily histiocytes. Acid-fast bacilli (AFB) staining would be negative in lymphoma.
**B.** Sarcoidosis – Incorrect. Sarcoidosis can present in the skin in up to 30% of patients, and occurs most frequently on the face, upper back, extremities, and sites of prior injuries.^1^^,^^2^ On histology, sarcoidosis is characterized by naked granulomas and AFB stain would be negative.
**C.** Keloid scar – Incorrect. Extensive body surface area involvement despite no known prior trauma and the presence of a scaly border make keloids less likely. On histology, keloid scars have bundles of collagen and are not typically organized into granulomas. Again, AFB staining would be negative.
**D.** Leprosy – Correct. Leprosy remains endemic in the coastal southeastern United States and Hawaii.^2^ This patient’s findings suggest histoid leprosy, a rare subtype of multibacillary (>5 skin lesions) lepromatous leprosy that presents with soft, shiny, flesh-colored to yellow-red dermal and subcutaneous papules to nodules favoring the extremities, buttocks, and lower back.^2^^,^^3^ Histoid leprosy can arise *de nov*o but is associated with resistance to dapsone monotherapy in the treatment of leprosy.^2^, ^3^, ^4^, ^5^ Lesions constitute a reservoir of *Mycobacterium leprae* and are extremely contagious.^3^ As demonstrated, biopsy of a lesion demonstrates granulomatous inflammation with positive AFB staining of organisms.
**E.** Leishmaniasis – Incorrect. The clinical presentation of keloidal plaques may be seen in diffuse cutaneous leishmaniasis but that is typically seen in immunocompromised patients. Histology of leishmaniasis may resemble that of lepromatous leprosy on hematoxylin-eosin stain. However, in the early stages of leishmaniasis, amastigotes are typically visible within the histiocytes and AFB staining would be negative.

**Question 2: What stain is expected to be positive on pathologic examination?**

**A.** CD3
**B.** AFB
**C.** CD34
**D.** Factor XIIIa
**E.** Van Gieson

**Answers:**

**A.** CD3 – Incorrect. CD3 is a pan-T-cell marker that differentiates T- vs B-cell lymphomas.^1^^,^^4^
**B.** AFB – Correct. This patient had AFB showing numerous organisms and polymerase chain reaction specific for *M leprae.* Diagnosis of leprosy is established through skin biopsy; gram, Ziehl-Neelsen, or Fite-Faraco stain demonstrating bacilli; and polymerase chain reaction to differentiate *M leprae* from other mycobacteria and confirm the species.^1^ AFB can be negative in some cases as *M leprae* have a thinner wall; thus, Fite stain, which contains an oil component, should be done to increase sensitivity if initial AFB is negative. Characteristic histopathological findings in histoid leprosy are fibrous nodules, globi (amphophilic collections of mycobacteria), and dermal expansion of histiocytes in a whorled and storiform pattern with no signs of foreign material.^3^, ^4^, ^5^ Additionally, a Grenz zone (band of normal-appearing dermis separating the epidermis from an infiltrate of plasma cells, lymphocytes, and Virchow cells) is characteristic of lepromatous leprosy and visualized on biopsy.^4^^,^^5^
**C.** CD34 – Incorrect. CD34 can be positive in various spindle cell tumors and can be helpful in differentiating dermatofibrosarcoma protuberans (DFSP), which would stain positive, from dermatofibroma, which is expected to be negative.^4^ DFSP is not part of the differential.
**D.** Factor XIIIa – Incorrect. Factor XIIIa is positive in dermatofibroma but not in DFSP.^4^ DFSP is not part of the differential.
**E.** Van Gieson – Incorrect. Verhoeff-van Gieson stains collagen and elastic fibers and can rule out smooth muscle tumors.^4^ It would be positive in keloids; however, histoid leprosy is more likely.

**Question 3: What are the next steps in management?**

**A.** Class I topical corticosteroids
**B.** Surgical excision of lesions
**C.** Reassurance
**D.** Intralesional steroid injection
**E.** Systemic antibiotics

**Answers:**

**A.** Class I topical corticosteroids – Incorrect. Topical corticosteroids are not indicated for the diagnosis in this case.
**B.** Surgical excision of lesions – Incorrect. Surgical excision is not a mainstay of leprosy treatment.
**C.** Reassurance – Incorrect. Given the contagious nature of histoid leprosy and the potential for disease progression and disfigurement, reassurance is not appropriate.
**D.** Intralesional steroid injection – Incorrect. Intralesional steroids is not a recommended treatment for this type of leprosy. It is used for treatment of nerve function impairment in the setting of leprosy. This patient did not have nerve involvement from his leprosy.
**E.** Systemic antibiotics – Correct. The patient was started on a multibacillary-multidrug therapy regimen of rifampin 600 mg monthly, moxifloxacin 400 mg monthly, minocycline 100 mg monthly, and vitamin D 50,000 units monthly for a total of 24 months. The patient had marked improvement with flattening of nodular lesions a few months after initiation of therapy. Though there is no current consensus on treatment of histoid leprosy, the World Health Organization recommends treating multibacillary disease (>5 lesions) with a 12 to 18-month regimen of rifampin 600 mg once monthly, clofazimine 300 mg once monthly and 50 mg daily, and dapsone 100 mg daily, followed by a 5-year observation period.^1^ Patients are no longer infectious after the first dose of treatment. Recommended laboratory monitoring includes baseline complete blood count and platelets, aspartate aminotransferase and alanine aminotransferase, calcium, glucose-6-phosphate dehydrogenase, blood urea nitrogen, creatinine, and bilirubin; complete blood count and platelets 1 to 2 months following treatment initiation; and repeat complete blood count, platelets, and aspartate aminotransferase/alanine aminotransferase every 3 months thereafter.^2^

## Conflicts of interest

None disclosed.
